# Case report: Pancreatic metastasis from small-cell lung cancer appears as primary G2 pancreatic neuroendocrine tumor on combined contrast PET imaging with three probes

**DOI:** 10.3389/fonc.2024.1403260

**Published:** 2024-10-16

**Authors:** Huimin Zhang, Jie Gao, Xiaofeng Cong, Chen Chen, Jiaxin Yin, Wenji Xiong, Ziling Liu

**Affiliations:** ^1^ Cancer Center, The First Hospital of Jilin University, Changchun, China; ^2^ Department of Radiology, The First Hospital of Jilin University, Changchun, China

**Keywords:** small-cell lung cancer, pancreatic metastasis, primary pancreatic neuroendocrine carcinoma, PET/CT, differential diagnosis, multiple primary cancers

## Abstract

Pancreatic metastasis is a rare malignant tumor; when it comes to multiple cancers, it may be a challenge to identify the primary lesion of new pancreatic metastases. With the continuous advancement of imaging technology, the PET/computed tomography (CT) has been widely used because of its high diagnostic accuracy and non-invasiveness. However, in the present case, the patient had history of limited small-cell lung carcinoma and prostatic cancer; the combined application of the three kinds of PET/CT was used to identify the new metastases of pancreatic and bone metastases, which suggested a high probability of primary G2 pancreatic neuroendocrine tumor with bone metastases. After the needle biopsy, samples were confirmed by diagnostic pathology as small-cell lung cancer metastasizing to the pancreas and bone. The results of our case suggests the irreplaceability of pathology and possibility of misdiagnosis by PET/CT; moreover, it also supplements clinical data for second primary cancers after small-cell lung cancer.

## 1 Introduction

Lung carcinoma rarely metastasizes to the pancreas, which accounts for approximately 0.6% of metastatic lung cancer. Small-cell lung cancer (SCLC) is the most common histological type of lung carcinoma metastasizing to pancreas, because it has a strong propensity to metastasize (1). Due to the special anatomical location of the pancreas and the high rate of complications of needle biopsy, therefore, the commonly used diagnostic criteria for pancreatic lesions is mainly based on clinical history and radiologic imaging. With the development of nuclear medicine diagnostics and treatment, the application of multi-tracer imaging modalities provided a strong basis for the precise diagnosis and effective treatment of malignant tumors; especially in cases where pathology acquisition conditions are limited, it can provide reference information for physicians. However, when it comes to the pancreatic metastases, which belongs to endocrine organ, it will be challenging to use the metabolic imaging of PET/computed tomography (CT) for differential diagnosis, and there will be a possibility of misdiagnosis; hence, the gold standard for diagnosis is confirmed by histology or cytology.

In this case, we will report on a patient with metachronous multiple primary cancers of SCLC treated 5 years ago and prostate cancer 1 years ago. The patient underwent 18F-fluoro-2-deoxy-2-D-glucose (18F-FDG) PET/CT because of bone pain, and it revealed space-occupying lesion in pancreas and left acetabulum, which was considered to be both metastatic cancers. In order to identify the primary lesion, he successively underwent examination of 18F-PSMA-1007 PET/CT and 18F-NOTA-JR11 PET/CT. By comparing tumors uptake of three different tracers, the result indicated a high possibility of primary grade 2 pancreatic neuroendocrine neoplasm; then, we performed endoscopic ultrasound-guided fine-needle aspiration biopsy and arrived at a final diagnosis of pancreatic metastasis and bone metastasis of SCLC. This report illustrates the complex diagnostic process and describes a very rare case of pancreatic metastasis of small-cell lung carcinoma, which may provide a valuable reference for the diagnosis of this type of patient. Moreover, it emphasizes the irreplaceability of pathology and the non-specific on PET/CT of pancreatic metastasis.

## 2 Case report

**Figure 1:**
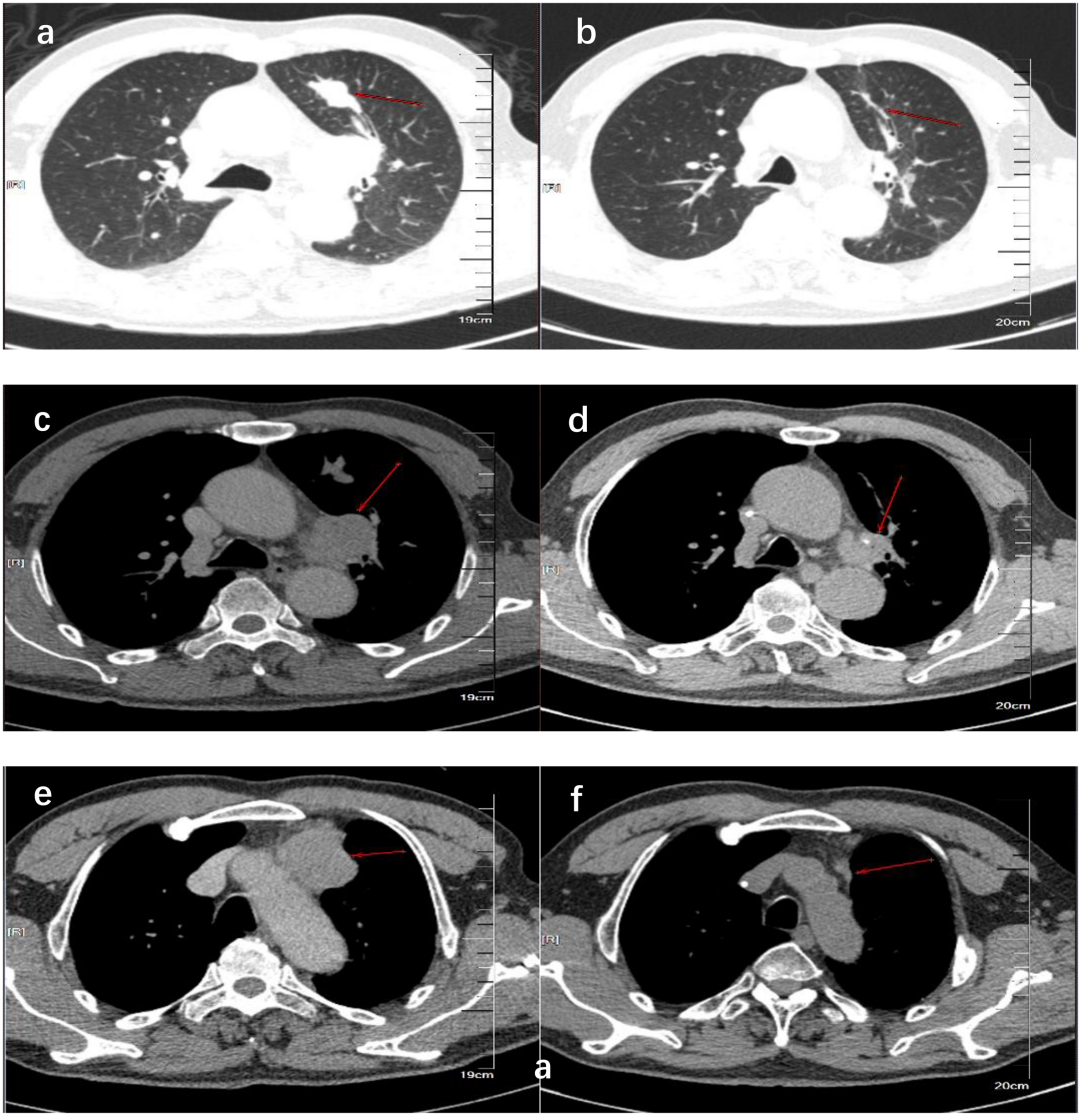
CT showed the comparison of lung lesions before **(A, C, E)** and after **(B, D, F)** the treatment of concurrent chemoradiotherapy. **(A, B)** show the tumor location of the primary lesion in the left upper lobe before and after treatment. **(C, D)** show the location of the tumor before and after treatment of the left hilar lymph node metastases. **(E, F)** show the tumor location before and after treatment of anterior mediastinal lymph node metastases.

We present the case of a 69-year-old man with a history of 20 pack-years of smoking, who underwent CT scan due to 4 months of a dry cough under no obvious inducement on 14 December 2018. The CT revealed a dense mass in the upper lobe of the left lung which measured approximately 2.8 cm × 1.5 cm in size; moreover, there were soft-tissue density lesions in the anterior mediastinum and left hilar area, which sized about 4.3 cm × 3.1cm and 3.4 cm × 3.0 cm, respectively (**Figures 1A, C, E**). The result of bronchoscopic transbronchial lung biopsy supported SCLC based on the immunohistochemistry analysis that revealed a positive staining for pan-cytokeratin (CKpan), thyroid transcription factor 1 (TTF-1), protein phosphatase 1 (Ki-67) (approximately 90%), synaptophysin (Syn), and cluster of differentiation 56 (CD56) and a negative staining for Tumor prot ein 63 (P63), Cytokeratins (CKs) 5 and 6 (CK5/6), and cytokeratin (CK7). Therefore, he was diagnosed with limited-stage SCLC and treated with concurrent cisplatin and etoposide chemotherapy combined with thoracic radiotherapy and standard prophylactic brain radiation. The last time for treatment was 17 July 2019, and the CT scan reported that the lesions size was about 1.1 cm × 0.5 cm in the upper lobe of the left lung and 2.0 cm × 1.3 cm and 1.9 cm × 1.8 cm in the anterior mediastinum and left hilar area, respectively (**Figures 1B, D, F**). Subsequently, the patient had regular follow-up examinations.

The patient was admitted on 3 March 2022 because his prostate-specific antigen (PSA) continued to rise over 6 months. Further magnetic resonance imaging (MRI) showed uneven signal in the central gland with Prostate Imaging Reporting and Data System (PI-RADS) assessment category 5, and the signal in the peripheral zone was reduced with PI-RADS 2. Targeted prostate biopsy was performed, which identified prostate adenocarcinoma component within the lesion. Therefore, he underwent robot assisted laparoscopic radical prostatectomy and abdominal mass resection. Postoperative pathology confirmed adenocarcinoma of the prostate and seminal vesicles. The Gleason score was 4 + 4; moreover, it also contained a small amount of Gleason 5 component (<5%). The tumor accounted for approximately 50% of the total prostate volume, involving bilateral lobes of the prostate and periprostatic adipose tissue; in addition, the infiltration of blood vessels, nerves, and lateral resection margin of prostate urethra could be seen. The immunohistochemistry analysis revealed a positive staining for Ki-67 (approximately 10%), PSA, P504S, and Syn and a negative staining for P63, PCgA, and 34βE12. Therefore, he was diagnosed with stage pT3aN0M0, and the administration of Flutamide started in May 2022, in which the dose was 750 mg/day, divided into three doses orally.

**Figure 2:**
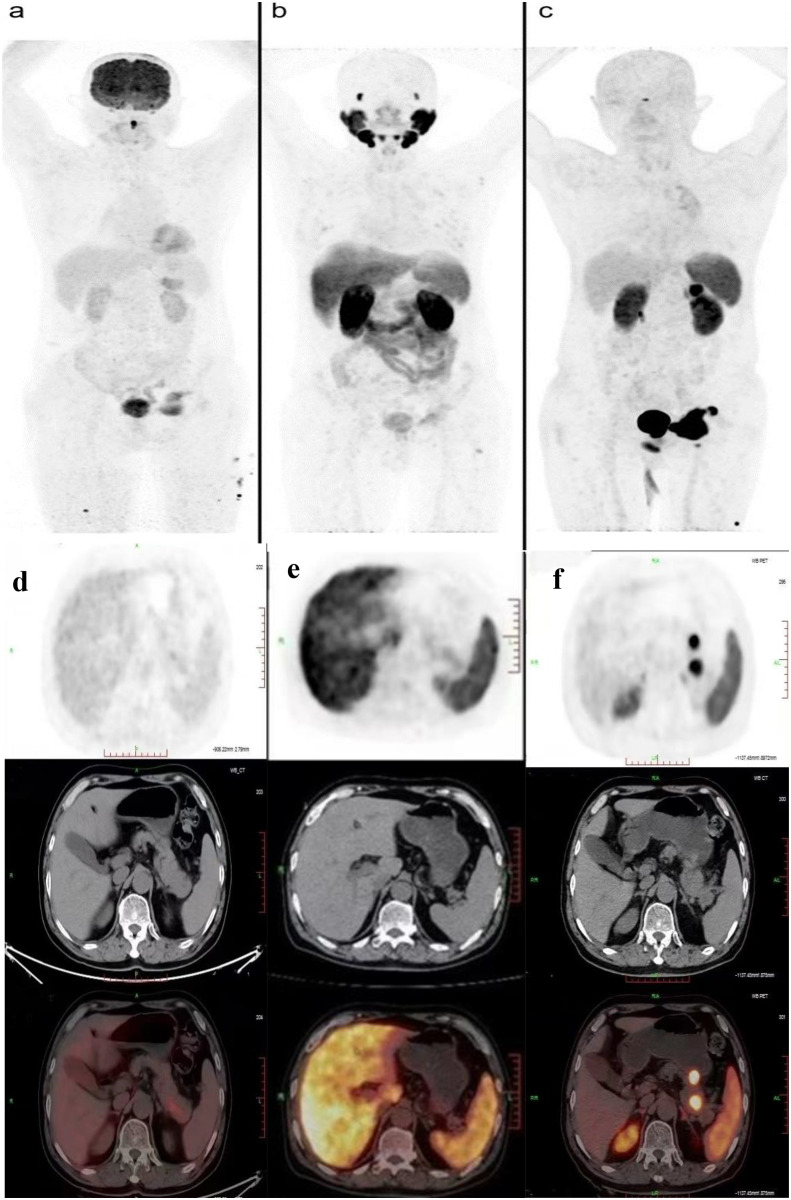
PET/CT showed comparison of the new lesions using 18F-FDG **(A, D)**, 18F-PSMA **(B, E)**, and 18F-JR11 **(C, F)** tracers.

**Figure 3:**
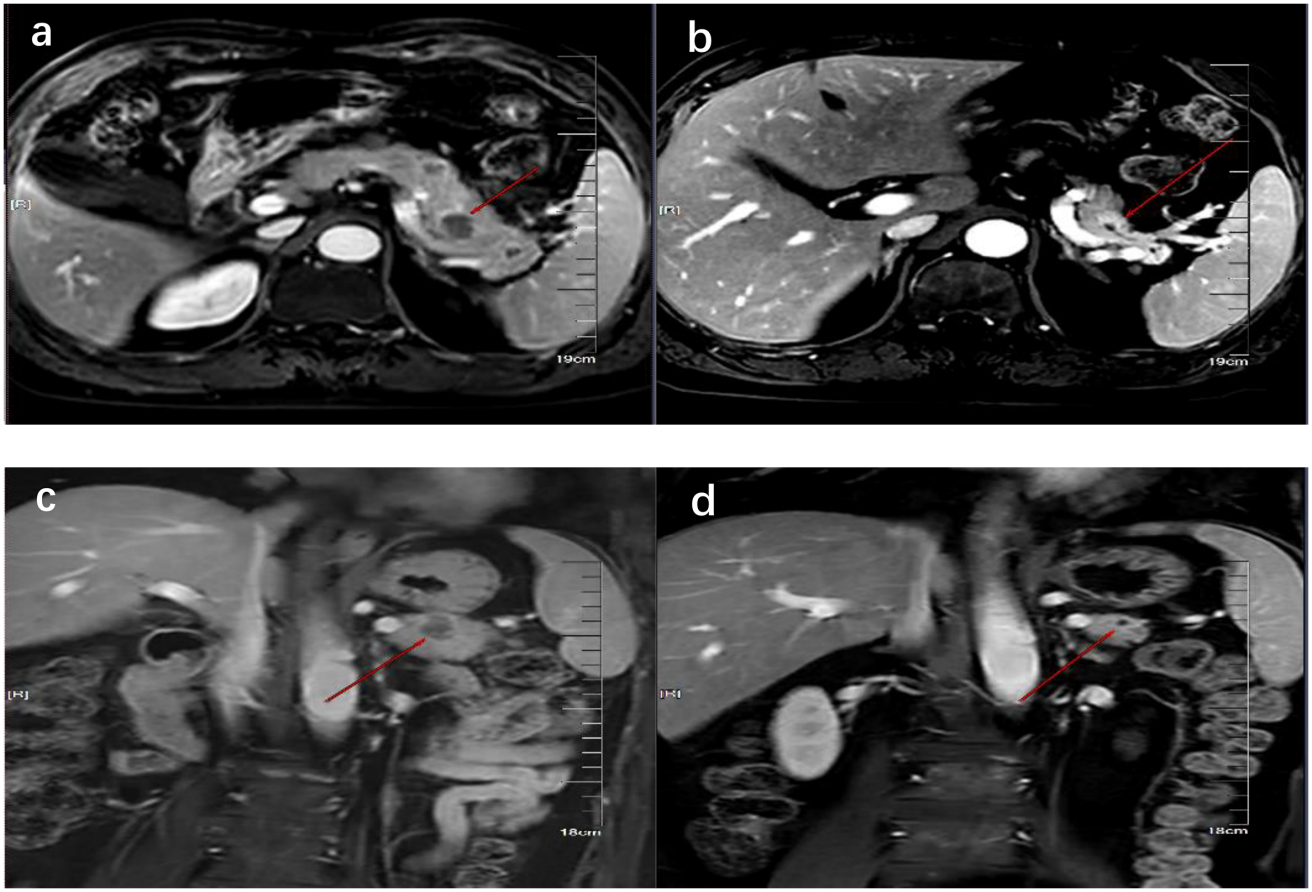
MRI showed the comparison of pancreatic lesions before **(A, C)** and after **(B, D)** the treatment of chemotherapy. **(A, B**) are MRI images of horizontal pancreas before and after treatment, and **(C, D)** are MRI images of coronal pancreas before and after treatment. The red line arrows point to the location of pancreatic lesions before and after treatment.

**Figure 4:**
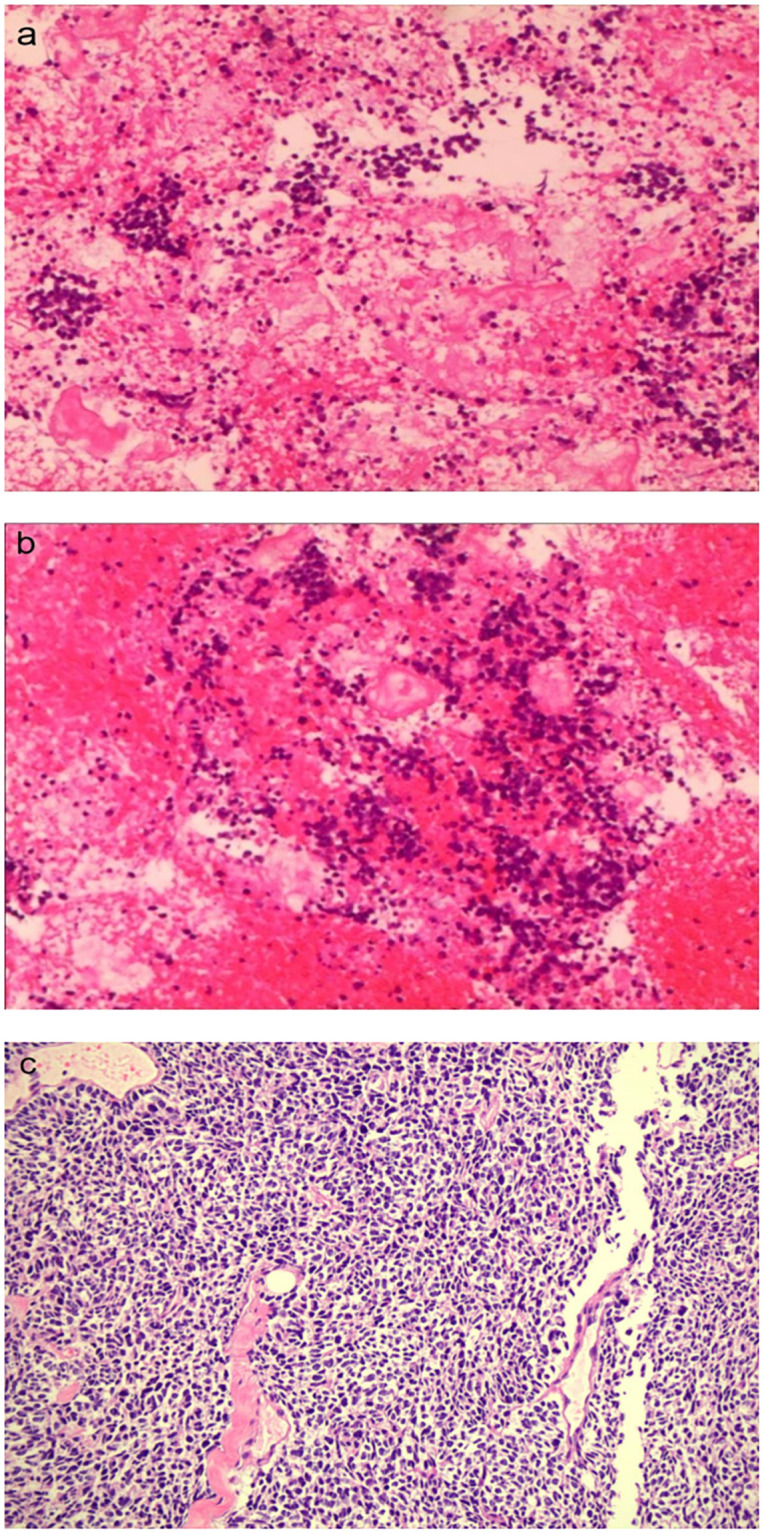
The pathological images of the pancreatic **(A, B)** (×100) and bone metastases **(C)** (×10), respectively.

On 6 October 2023, he underwent 18F-FDG PET/CT for bone pain, which demonstrated that the dense streak shadow with low glucose metabolism in the upper lobe of the left lung was consistent with change after treatment and tumor activity was inhibited. Moreover, the presentation of PET was also consistent with postoperative manifestation of prostate cancer, and no abnormal hypermetabolic lesions were found in the surgical area. Hypermetabolic activity and osteolytic bone destruction sized about 3.8 cm × 3.2 cm were distinguished in left acetabulum lesion, which was considered bone metastasis (SUV max = 7.3). In addition, there were lesions with hypermetabolic activity in the pancreas, which was also supposed to be metastases (SUV max = 4.2) (**Figures 2A, D**), and the largest one sized about 5.1 cm × 2.8 cm on MRI (**Figures 3A, C**). The conclusion of 18F-PSMA-1007 PET/CT was in accordance with 18F-FDG PET/CT (**Figures 2B, E**). Then, 18F-NOTA-JR11 PET/CT was performed to compare with 18F-PSMA-1007 PET/CT and 18F-FDG PET/CT, which showed multiple hypermetabolic lesions in the head and tail of the pancreas, exhibiting an abnormally high 18F-NOTA-JR11 uptake (SUV max = 17.6) (**Figures 2C, F**) but a moderate 18F-FDG and 18F-PSMA-1007 uptake. Therefore, the high possibility of G2 grade primary neuroendocrine tumors (NETs) of the pancreas was considered. Moreover, the lesion in the left acetabulum presented the similar pattern of uptake (SUV max = 31.9), which was considered pancreatic NET with bone metastasis. The serum levels of tumor markers were as follows: Carbohydrate antigen 199 (CA199) of 33.68 U/mL and Neu-ron specific enolase (NSE) of 23.74 ng/mL. To identify the primary lesion, we performed endoscopic ultrasound-guided fine-needle aspiration biopsy and percutaneous puncture under ultrasound guidance for bone biopsy. The result of bone puncture biopsy supported small-cell carcinoma, which was likely to originate from the lung based on the immunohistochemistry and morphology analysis, and immunohistochemistry results were a positive staining for Ki-67 (approximately 80%), CKpan, TTF-1, CgA, Syn, Insulinoma-associated protein 1 (INSM1), Retinoblastoma gene product (RBGP), and P53 (approximately 80%) and a strongly positive staining for SSTR2; simultaneously, it indicated a negative staining for NapsinA, P504S, PSA, Vimentin, CK7, P40, and NKX3.1 (**Figure 4C**). For the tissue of pancreas, abnormal cells were found in the coagulation tissue, which supported the diagnosis of small-cell neuroendocrine carcinoma (NEC), and immunohistochemistry analysis showed a positive staining for Ki-67 (approximately 40%), P53, CK, Syn, CD56, CK19, INSM1, and alpha-thalassemia mental retardation X-linked (ATRX) and a negative staining for CK7, CK20, villin, CEA, CgA, and RbGP (**Figures 4A, B**). In the meanwhile, pancreas tissue sample was obtained, and a comprehensive genomic profiling was performed by high-throughput sequencing using a 520 cancer-related gene panel, which demonstrated RB1 p.Y454 and TP53 p.G266R variant (germline mutation). The treatment consisted of cisplatin + etoposide + Atezolizumab + denosumab starting on 15 November, and, after 2 courses of treatment, the overall efficacy was evaluated as partial response, and the largest pancreas lesion size was about 3.4 cm × 1.5 cm (**Figures 3B, D**).

## 3 Discussion

In this paper, it was difficult to determine the nature of the lesions because the patient had a history of malignant tumors including SCLC and prostate cancer, which both have a strong predilection to metastasize to bone. SCLC is characterized by its extremely aggression and ability to metastasize to a variety of anatomic sites such as the bones, liver, and brain (2), with a 27%–41% incidence of bone metastases (3). Meanwhile, bone is also a preferred site for prostate cancer cells; approximately 5%–10% of newly diagnosed prostate cancer patients showed evidence of bone metastasis (4, 5); therefore, bone space-occupying lesions may be secondary metastases of one of the two malignant tumors mentioned above. On the other hand, whether lung cancer or prostate cancer, it is considerably rare to spread to the pancreas. Preliminary PET/CT results revealed that no increased 18F-FDG uptake was seen in the lungs or prostate, whereas pancreatic and bone lesions were likely to be metastases. Therefore, it is important to identify the primary tumor in metastatic disease. Moreover, it is necessary to clarify whether the pancreas and bone lesions belonged to the same pathological type. Although pathology diagnosis is known as the gold standard for disease diagnosis, the pancreas is a retroperitoneal organ and it has a deep anatomical position with complicated surrounding structures; biopsies could be painful and carry risks of complications, toward which patient had negative attitudes. With the rapid development of computer technology and medical imaging technology, PET/CT imaging has been widely recognized in clinical practice for diagnosis and clinical staging for the advantages of functional and anatomical structural imaging as well as non-invasiveness, which has become an important part of precision medicine. To respect patient wishes and to avoid as much as possible the use of an invasive operation, thereby, we wanted to make a further diagnosis through imaging technology. Considering the patient was diagnosed with prostate cancer last year, therefore, it is necessary to firstly identify whether new lesions originated from the prostate. Nowadays, prostate-specific membrane antigen (PSMA) PET/CT is an emerging imaging method, which is becoming a promising method for staging in prostate cancer (6). PSMA, a type II transmembrane glycoprotein, is highly overexpressed in prostate cancer epithelial cells, which was 100–1,000 times higher than that in normal cells (6, 7). Moreover, PSMA expression increased incrementally with increasing cancer stages and tumor grades in prostate cancer, especially in high-grade, metastatic, and castration-resistant disease (8). Therefore, PSMA is an excellent target for specific imaging and targeted therapy for prostate cancer; with 18F-PSMA-1007 PET/CT, disease recurrence could be identified at a low level of serum PSA (9). The patients underwent PET with 18F-labeled PSMA ligands, aiming to determine whether there was evidence of progressive prostate cancer, whereas imaging test identified no increased metabolic activity in prostate cancer post-operative area; meanwhile, the mean SUV of the bone and pancreas lesion reported was slightly high which were 4.9 and 7.5, respectively. Considering the patient’s PSA was within the reference value, we estimated that new lesions were less likely to be metastasized from prostate cancer.

Considering the history of SCLC, therefore, another non-invasive method to verify the possibility of pulmonary source was urgently needed. To meet the needs of clinical treatment and diagnosis, promising imaging technology on the basis of specific biological targets to cancer are being explored. Somatostatin receptor (SSTR) is overexpressed on the majority of neuroendocrine neoplasms cell surface, and somatostatin receptor subtype 2 (SSTR2) has become an essential target for diagnosis and radionuclide therapy of neuroendocrine neoplasms (10). However, the expression of SSTR in tumors is not limited to NETs and can also be found in a variety of other solid tumors, including SCLC cancer (11). JR11 was recently developed as an SSTR2-specific antagonist for PET tracer, which can bind to significantly more receptor sites than the SSTR agonists, showing a more favorable pharmacokinetics, better contrast, and better lesion detection rate (12, 13). Currently, 18F-NOTA-JR11 PET/CT imaging has a huge potential in clinical practice for identifying the primary lesion, clinical staging, and restaging of neuroendocrine neoplasms (11, 14, 15). What is noteworthy is that neuroendocrine neoplasm gathers a heterogeneous group of tumors with histological, distinct clinical, and genetic characteristics, which is classified into well-differentiated NETs and poorly differentiated NECs. SSTR2 positive is significantly lower in poorly differentiated than in well-differentiated neuroendocrine neoplasm, whereas 18F-FDG uptake has been proven to be significantly higher in poorly differentiated than in well-differentiated neuroendocrine neoplasm (16). In NECs, 18F-FDG uptake is usually increased and about half of cases have increased SSTR-PET uptake; therefore, it will be significantly higher in sensitivity of differential diagnosis when using dual-tracer PET/CT (18F-FDG and 18F-NOTA-JR11) than FDG/PET imaging alone (17, 18). The patient received a 18F-NOTA-JR11 PET/CT, and the image showed a high uptake of 18F-NOTA-JR11 in pancreatic occupying lesion and bone lesion, which suggested a possible G2 grade primary pancreatic NET with bone metastasis. Given the patient’s prior history of SCLC and multiple primary cancers, we tended to think that new lesions were more likely to be metastasized from SCLC when it comes to the new neuroendocrine neoplasm of the pancreas. Accurate differential diagnosis of neuroendocrine neoplasm is important for treatment selection and assessment of prognosis, and it was difficult to identify the differentiation level based on imaging data alone. Comprehensive communication with patient was conducted, and he finally consented to undergo biopsy. Therefore, we performed biopsy of pancreatic lesions by endoscopic ultrasound-guided fine-needle aspiration and percutaneous biopsy of bone lesions. The diagnosis of bone lesion was clear based on pathology and immunohistochemistry, which was confirmed a bone metastasis from SCLC. In addition, SSTR2 expression levels (score 3+) in tumor tissue explained the reason why uptake of 18F-NOTA-JR11 increased in the lesions, increasing difficulty diagnosis. The differentiation between neuroendocrine tumor G3 and neuroendocrine carcinoma is challenging due to the limited sample tissue available from pancreatic lesions, constraining the reliability of current morphological and immunohistochemical criteria. Therefore, the immunohistochemical staining of TP53, RB1, and ATRX was conducted to assist in differential diagnosis. The expected immunohistochemical phenotype is usually TP53(−), RbGP(+), and ATRX(+/−) in well-differentiated NETs but TP53(+), RbGP(−), and ATRX(+) in poorly differentiated NECs (19). The report was TP53(+), RbGP(−), and ATRX(+) in our case; therefore, he was definitively diagnosed with SCLC developing pancreatic metastasis. For lung cancer patients with pancreatic and bone metastases, a comprehensive treatment based on systemic therapy is the main principle for them. Two recent independent large-scale phase III clinical studies of CASPIAN and IMpower133 have provided robust evidence that immunotherapy plus chemotherapy can extend the overall survival of patients with extensive stage SCLC (20, 21). The patient experienced relapse beyond 6 months after the first-line therapy, and no immunotherapy has been used in the first line; therefore, he was treated with the treatment regimen including Etoposide plus Cisplatin plus Atezolizumab; simultaneously, Denosumab was used for bone metastases. The patient tolerated the treatment well, and, after two courses of treatment, the lesion was significantly smaller than before, which confirmed the accuracy of our diagnosis. This case emphasizes the unsubstitutability of pathology. Although the three imaging probes—18F-FDG, 18F-PSMA-1007, and 18F-NOTA-JR11—were combined, diverse imaging equipment, imaging mode, and, more importantly, receptor expression in tumor cells may lead to many differences in diagnosis results.

In addition to the rarity of metastatic sites, what is also rare in this case is that patient developed a second primary cancer 4 years after successful treatment for limited-stage SCLC. Given the highly aggressive nature of small cell lung carcinoma (SCLC), a subtype of lung cancer known for its poor prognosis, the five-year relative survival rate stands at approximately 10% to 13% (22). As a consequence, little attention has been paid to second primary cancers after SCLC. However, with the development of comprehensive anti-tumor treatment, the prolonged survival outcomes increased risk of developing metachronous second primary malignancies. Previous research predicted that the incidence rate of second primary malignant tumors after limited-stage SCLC treatment is approximately 2.8% (23). Given the special nature of the case, the genetic testing was completed, which identified somatic mutations in TP53 and RB1. TP53 and RB1 are widely believed to be tumor-suppressor genes in multiple tumors, which plays an important role in regulating cell division (24, 25). In the meanwhile, inactivation of tumor suppressor genes of TP53 and RB1 is common in almost all cases of SCLC (26). These mutational loads may promote the development of second primary cancer. Future studies on comprehensive molecular analysis may shed more light on underlying mechanisms of these tumor-suppressor genes in the development and progression of SCLC and second primary cancer.

In this article, we report the diagnostic process of a rare case of multiple primary cancers with SCLC metastasizing to pancreas and bone. The patient developed metastases five years after successful treatment for limited-stage SCLC and 1 year after prostate cancer. After combining the three imaging probes—18F-FDG, 18F-PSMA-1007, and 18F-NOTA-JR11, the imaging characteristics of pancreatic lesion on PET/CT were consistent with G2 grade primary pancreatic NET, However, it was confirmed by pathology that malignant cells were SCLC. Indeed, the past medical history of multiple primary cancers poses a major challenge to our differential diagnosis; in addition, the presentation of the combined three imaging probes was inconsistent with our pathological diagnosis because the diverse molecular biology may cause the expression of some special cancer phenotype, which will interfere diagnosis; therefore, it should alert clinicians to the need for pathological diagnosis. We use a variety of tracers to improve diagnostic sensitivity and resolution; however, different radiopharmaceuticals present a higher radiation exposure to patients, which is a reminder that we can reduce the use of radiopharmaceuticals with the help of whole-body PET/CT to reduce radiation exposure (27, 28). Moreover, our case enrich data of the second primary cancer after SCLC; although genetic testing studies have been performed, additional research are needed to identify the critical role of TP53/RB1 in the second primary cancer. Clearly, future studies will unravel the underlying molecular mechanisms in more detail, bringing us closer to precision medicine.

## Data Availability

The original contributions presented in the study are included in the article/supplementary material. Further inquiries can be directed to the corresponding authors.
